# Dual Screen for Metal-Tolerant Metallophore Producers Evaluated with Soil from the Carpenter Snow Creek Site, a Heavy-Metal-Toxified Site in Montana

**DOI:** 10.1021/acsomega.4c07306

**Published:** 2024-12-16

**Authors:** Mohammed M. A. Ahmed, Cameron Hammers, Paul D. Boudreau

**Affiliations:** † Boudreau Lab, Department of Biomolecular Science, School of Pharmacy, University of Mississippi, Faser Hall University, University, Mississippi 38677-1848, United States; ‡ Department of Pharmacognosy, Al-Azhar University, Nasr City, Cairo 11371, Egypt

## Abstract

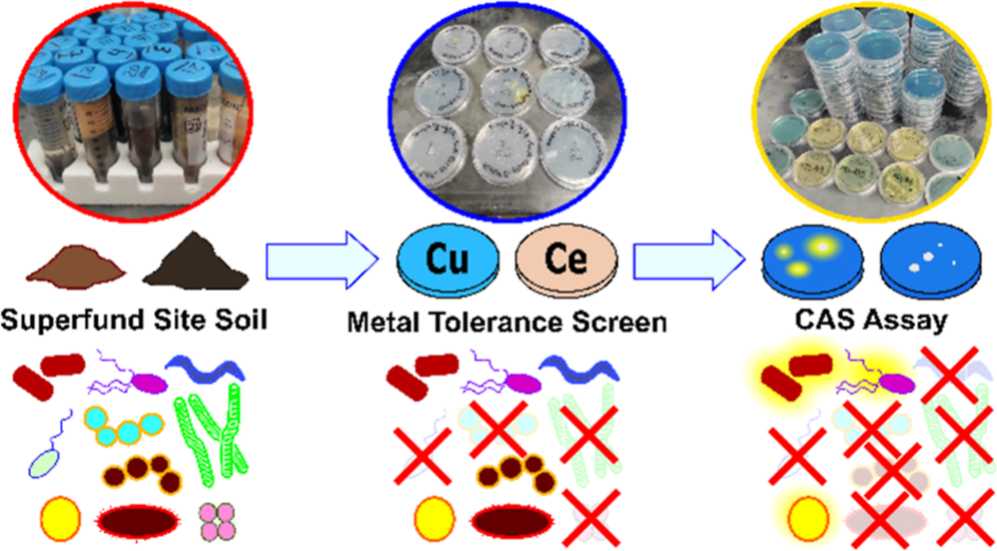

Bacteria have evolved numerous mechanisms to resist metal toxicity, including small-molecule metal chelators (metallophores). This study presents a dual screening methodology to isolate metallophore-producing bacteria from the Carpenter Snow Creek Mining District for potential use in heavy-metal bioremediation. Soil samples were screened on metal-supplemented plates from which colonies were picked onto chrome azurol S (CAS)-dyed plates. Copper or cerium toxicity was used as the primary selection step, while the CAS assay revealed the excretion of metal-binding compounds. From the pool of bacteria encompassed in the native soil microbiome, fifty-one isolates were picked from metal-toxified media by colony morphology. Out of these colonies, 17 exhibited positive results in the CAS assay. 16S rRNA sequencing identified eight unique species within these CAS-positive hits, the nearest BLAST hits of which were from the genera: *Rhodanobacter*, *Dyella*, *Bradyrhizobium*, *Luteibacter*, *Cupriavidus*, *Arthrobacter*, and *Paraburkholderia*. To validate our workflow, we profiled our *Cupriavidus* isolate by LCMS metabolomics and genome mining and purified its metabolites. These efforts led to the reisolation of the known metallophore taiwachelin. In efforts to identify lead strains for heavy-metal bioremediation applications, the present work suggests the utility of our screening method in rapidly targeting the metallophore producers from the soil microbiome.

## Introduction

Heavy-metal pollution stands as a major environmental concern, with sources from the natural world, as well as industry, mining, and fossil fuel burning, all demanding immediate and concerted action to mitigate their deleterious effects on both the environment and humans.^1−3^ The impact on human health is particularly concerning, as heavy metals such as lead, mercury, and cadmium have well-characterized toxicities in the body.^4−7^ Heavy metals can damage organelles such as mitochondria, lysosomes, and the cell membrane, as well as enzymes, DNA, and nuclear proteins, leading to DNA damage, cell cycle disruption, apoptosis, or carcinogenesis.^8^ Long-term exposure to heavy metals can also disrupt the endocrine and immune systems, leading to chronic health issues.^9,10^

Yet, implementing traditional remediation techniques often proves economically burdensome, highlighting the need to seek cost-effective alternatives.^11−13^ Sustainable solutions such as bacterial bioremediation are good alternatives within which there are many strategies for addressing heavy-metal contamination.^11,14^ In the pursuit of harnessing their potential for bioremediation, dedicated research efforts have focused on the isolation and characterization of metal-tolerant bacteria (MTB) from a wide array of natural habitats.^15−18^ These efforts seek to uncover bacterial species endowed with specialized mechanisms tailored to withstand and neutralize the toxic effects of heavy metals such as the secretion of small-molecule metal chelators, commonly referred to as metallophores.^19−21^

Metallophores are molecules that exhibit high affinities for metal ions,^21−26^ they have proven roles in removing and detoxifying toxic metals.^22,25−27^ For example, deferoxamine B is a natural siderophore, originally discovered in a soil bacterium, *Streptomyces pilosus*, and it has been pharmaceutically used as an antidote to iron toxicity (Desferal) for decades.^28^ In addition to the ferric ion, more than 20 different metal complexes of deferoxamine B have been characterized.^29^ Moscatello and co-workers used a yersiniabactin, a metallophore produced by different Gram-negative bacteria,^23,30,31^ immobilized within a packed-bed column for continuous removal of copper and nickel from industrial wastewater.^32^ In a similar study, Ahmadi et al. used a heterologous biosynthetic system to produce yersiniabactin for the removal of a copper–zinc mixture from water.^33^ This prior work demonstrates the need for novel metallophores to expand these applications and expand the toolkit of small molecules available for environmental bioremediation.

Metal-binding small molecules are diverse and defy simple classification into groups like siderophores, a term specific to small molecules which assist in iron acquisition, which is often used exclusively from the metallophore label for metal chelators that aid in heavy-metal resistance.^23,26,34^ However, as we seek metallophores for bioremediation purposes, it is increasingly recognized that metallophores can fulfill multiple ecological roles.^21,24^ Siderophores are secreted by bacteria to chelate iron from the environment to make it bioavailable for the bacterial cell to grow and survive,^35,36^ but previous studies have shown siderophores with dual functions, such as delftibactin and yersiniabactin metallophores.^23,26,34^ Those two siderophores also possess the ability to chelate or biomineralize toxic noniron heavy-metal ions, e.g., copper or gold, in addition to their role in bacterial iron acquisition.^23,26,34^ These observations raise the possibility that other siderophores may have dual-role activities in heavy-metal resistance. Hypothesizing that in evolving dual-role functionalities, these metallophores may better bind heavy-metal pollutants, versus siderophores selective exclusively for iron, underpins our interest in these metallophores.

The soil microbiomes encompass a wide array of microbial communities.^37^ To isolate from soil microbiomes particular bacteria with specific functions, it is necessary to employ enrichment culture techniques that target desired microbes out of a larger community, such as the pioneering work on an aerobic nitrogen-fixing *Azotobacter* bacterium by Beijerinck.^38^ To that end, isolating MTB can be simply accomplished by using inhibitory concentrations of metals in various media to prevent the growth of susceptible microorganisms and enrich for MTB.^15−18^ Prior work has investigated heavy MTB, for applications such as plant growth-promoting characteristics (including siderophore production).^39^ Majewska and co-workers isolated siderophore producers, then tested these bacteria for their ability to bind to other metals.^40^ In this study, we wanted to enrich for MTB and then screen those bacteria for siderophore production, as any dual-role metallophore producers should be positive hits in both screens. Soil samples were obtained from a former mining site contaminated with heavy metals, the Carpenter Snow Creek Superfund National Priorities List Site; which was a producer of silver, lead, and zinc with residual tailings and low-grade ore also contaminated with copper.^41^ Initially, the soil microbiome was screened for MTB by using metal-treated plates. Copper was selected for its presence at our study site,^41^ while cerium was selected for its trivalent oxidation state which we hypothesized would lead to different small molecule–metal interactions than the divalent cupric ions. Cerium is also an inner transition metal, which is valuable to industry, has significant US supply risks, and for which novel recycling methods (such as metallophore-based techniques^24,32^) are needed.^43^ Subsequently, a secondary screen using the Chrome Azurol S (CAS) assay^42^ was employed to isolate only those MTB that produce metallophores.

## Results and Discussion

### Isolation of Metallophore-Producing Bacteria

Metallophore-producing MTB were isolated from the Carpenter Snow Creek soil by using our dual-screen method. In our effort to identify bacteria capable of surviving metal stress, MTB, we employed 1/5× diluted Luria−Bertani-Lennox (1/5 LB, Sigma-Aldrich), International Streptomyces Project medium 4 (ISP-4),^62^ or our lab’s Defined medium for Siderophores (DMS)^45^ supplemented with either copper or cerium. From these plates, a total of fifty-one distinct colonies were picked based on exhibiting unique morphological characteristics. Under the second screening step utilizing CAS-dyed plates of either 1/5 LB or DMS (note: colonies from the ISP-4 plates were tested for CAS activity on the 1/5 LB plates), only 17 of the original fifty-one colonies displayed a positive response in the CAS assay indicative of metallophore secretion. These identified CAS-positive hits were subsequently cultured in liquid media to prepare long-term frozen stocks, and bacterial isolates were given a strain code of BL-MT-01 through BL-MT-17.

### 16S rRNA Sequencing of the Isolates

**Figure 1:**
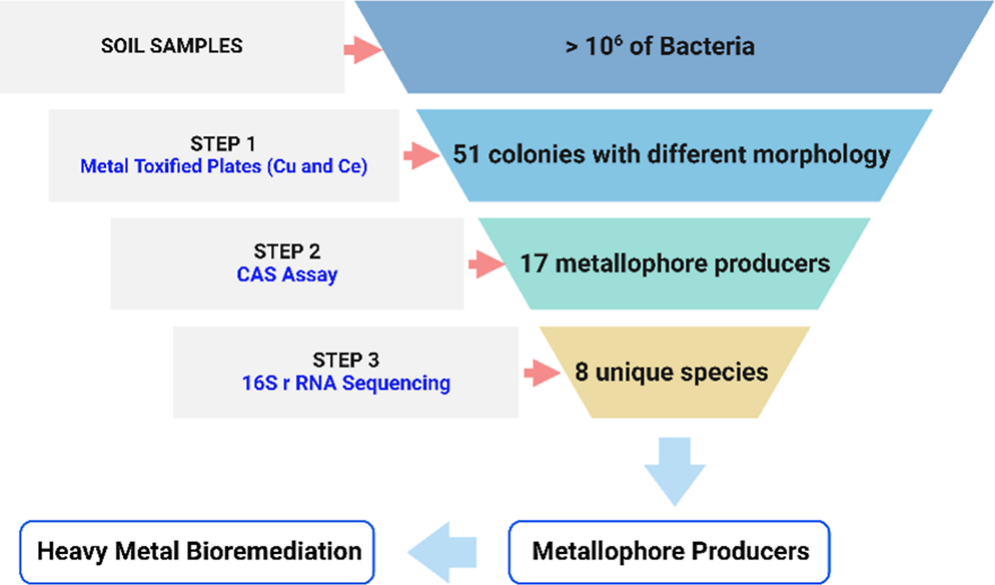
Dual screening method successfully targets metallophore-producing bacteria from the soil microbiome by substantially reducing the number of bacterial candidates for the desired application in heavy-metal bioremediation.

The bacterial isolates underwent 16S rRNA sequencing to identify the taxonomy of the isolates and to streamline the selection process for further investigation. Analysis of the 16S rRNA results using multiple sequence alignment unveiled that several isolates had identical sequences, which were presumed either to represent repeated isolations or the same species; though insufficient discrimination based on the 16S gene is also a possibility as has been observed using comparison to whole genome-based methods.^44^ Based on our 16S analysis, a representative strain of the eight distinct bacterial species was chosen, which was compared by BLAST to known sequences (Table 1 and Figure 1). The 16S sequences used were uploaded to GenBank under accession numbers PP868352–PP868368 (see Table S1 for the complete list).

**Table 1:** 16S rRNA-Based Taxonomic Identification of the Bacterial Isolates

strain code	accession number	nearest BLAST 16S hit (accession no.)	% similarity	read length	medium	metal
BL-MT-01	PP868352	*Paraburkholderia caledonica* LMG 19076 (NR025057)	98.6	844	DMD	CeCl_3_
BL-MT-06	PP868357	*Dyella ginsengisoli* Gsoil 3046 (NR041370)	99.6	247	ISP-4	CuCl_2_
BL-MT-07	PP868358	*Bradyrhizobium erythrophlei* CCBAU 53325 (NR135877)	100	1189	ISP-4	CuCl_2_
BL-MT-08	PP868359	*Rhodanobacter umsongensis* GR24-2 (NR108435)	98.2	1209	1/5 LB	CuCl_2_
BL-MT-10	PP868361	*Cupriavidus basilensis* DSM 11853 (NR025138)	99.9	928	DMD	CuCl_2_
BL-MT-11	PP868362	*Arthrobacter gyeryongensis* DCY72 (NR133699)	99.7	1167	1/5 LB	CeCl_3_
BL-MT-12	PP868363	*Luteibacter rhizovicinus* LJ96 (NR042197)	100	1216	1/5 LB	CuCl_2_
BL-MT-17	PP868368	*Paraburkholderia fungorum* LMG 16225 (NR025058)	100	1124	ISP-4	CuCl_2_

### Whole Genome Sequencing and Genome Mining of BL-MT-10

In our efforts to validate this dual screening methodology for targeting the metallophore producers, we selected the *Cupriavidus* strain, BL-MT-10, for further genome mining because of this genus’s known production of metallophores, such as cupriachelin or taiwachelin.^45,46^ The high molecular weight (HMW) DNA of the *Cupriavidus* strain was extracted and sequenced. The sequenced genome for this isolate was assembled into a circular chromosome of 4.47 Mb, a chromid of 3.72 Mb, and three plasmids with lengths of 40.6, 198, and 725 kb. This genome was compared against other members of the *Cupriavidus* genus via the OrhoANI tool (OAT)^47^ (see Figure S1) which showed a >96% similarity to both *Cupriavidus basilensis* strains DSM 11853 and 4G11.

Given this result, we have identified our strain as a member of this species, referred to throughout the rest of this report as *C. basilensis* BL-MT-10. The assembled genome of BL-MT-10 was mined for biosynthetic gene clusters (BGCs) that might potentially be encoded for metallophore production using antiSMASH.^48^ The result revealed a metallophore BGC with 88% similarity to the known taiwachelin pathway from *Cupriavidus taiwanensis* LMG19424^46^ on the 725 kb plasmid.

### LCMS-Based Metabolomics of *C. basilensis* BL-MT-10

To investigate if the identified homologue of the taiwachelin BGC in *C. basilensis* BL-MT-10 produced a similar molecule, we isolated and profiled the excreted metabolome of *C. basilensis* BL-MT-10 grown in DMS. *C. basilensis* BL-MT-10 metabolites were analyzed using the LCMS method our laboratory previously established for metallophore identification.^45^ The acquired data were scrutinized using the Global Natural Product Social (GNPS) platform.^49^ Within the data set, we primarily searched for the predicted [M + H]^+^ mass for the taiwachelin (963 *m*/*z*) to verify if the metabolomic profile of this bacterium matches the annotation of the metallophore BGC observed in the antiSMASH genomic mining. We identified a distinct cluster of masses exhibiting *m*/*z* values of 947, 963, and 991.

**Figure 2:**
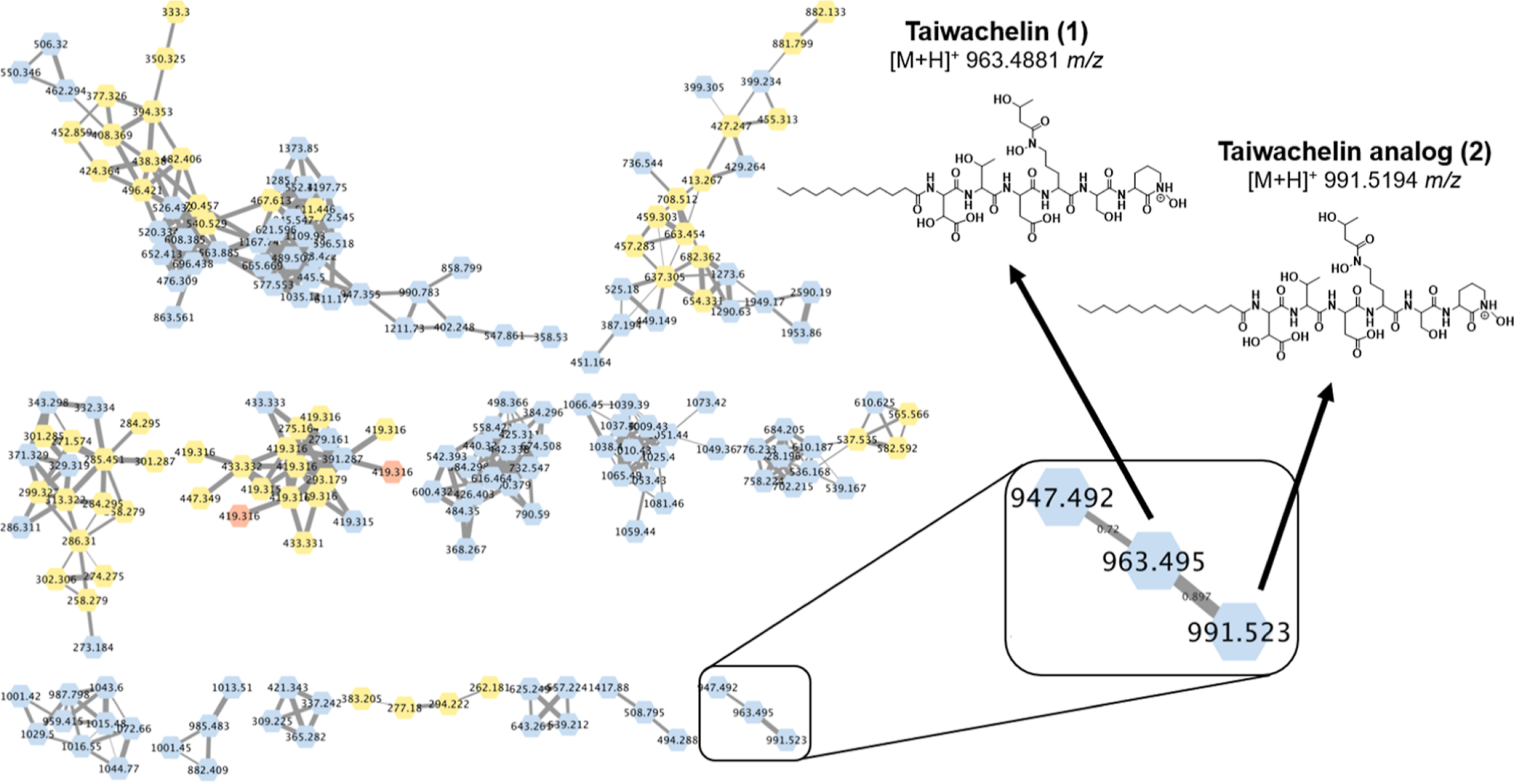
Metabolomic analysis of *C. basilensis* BL-MT-10 revealed a cluster of putative taiwachelins (highlighted section [M + H]^+^ of *m*/*z* 963 and 991 for taiwachelin (**1**) and its analogue (**2**), respectively). Nodes are colored by the samples in which they were observed: blue = supernatants from *C. basilenesis* BL-MT-10, red = blank runs, and yellow = both. Thicker lines indicate higher cosine scores.

To gain deeper insights into the structural characteristics of these compounds, we manually investigated the fragmentation spectrum of the 963 and 991 parent ions which were consistent with taiwachelin (**1**)^46^ and an analogue with a lipid-tail modification (**2**, see Figures 2 and S2–S5). This comprehensive approach, integrating metabolomic analysis with genomic insights, provided robust evidence supporting the secretion of metallophores by *C. basilensis* BL-MT-10, with taiwachelin lipopeptides identified as key candidates.

### Isolation of Taiwachelin Metallophore from *C. basilensis* BL-MT-10

The initial MS/MS fragmentation analysis suggested that the annotated metabolites included taiwachelin (**1**), a known metallophore reported not to bind copper.^46^ To obtain enough material for the isolation and characterization of this metallophore, we cultured BL-MT-10 at a 2 × 1 L scale using our DMS.^45^ The crude extract (ca. 200 mg) was fractionated via reversed-phase solid-phase extraction (RP-SPE), yielding 48 mg from the 50% MeCN fraction. This fraction was extensively purified using RP-HPLC, resulting in the isolation of compound **1** (20 mg). The isolated compound **1** was then subjected to NMR analysis and compared with previously reported data on taiwachelin. The NMR data of **1** showed agreement with the previously reported data for this molecule (see Table S2 and Figures S6–S8).^46^ This result was also consistent with our MS/MS fragmentation analysis (see Figures S2 and S3) and the high homology between the metallophore BGC in *C. basilensis* BL-MT-10 and the original producer, *C. taiwanensis* LMG19424.^46^ A crude assessment of the metal binding capacity of taiwachelin showed that when the pure compound was mixed with ferric iron, a stable complex formed, which could be detected as a 1016.3973 *m*/*z* [M – 2H + Fe]^+^ adduct (−1.7 ppm) using our LCMS method, suggesting strong binding in competition with the formic acid-acidified LCMS buffers. However, when mixed with Cu^2+^, Zn^2+^, or Ce^3+^ ions, any adducts that formed with taiwachelin were not stable to the LCMS conditions and only the native taiwachelin [M + H]^+^ adduct was detected, similar to prior results with this molecule.^46^

## Conclusions

With literature precedence to support the notion that metal-binding small molecules do not always fall into easily categorized groups such as siderophores to aid iron acquisition or metal chelators to aid in heavy-metal resistance but instead that metallophores can have multiple roles,^23,26,34^ we set out to develop a way to enrich strains which produce these molecules. Our dual-screen method selectively isolates bacteria capable of both resisting heavy-metal stress and producing siderophores, as this profile is what we hypothesize will be present in strains using metallophores to resist heavy-metal toxicity from the larger pool of the soil microbiome. Previous research has focused on isolating MTB using media supplemented with toxic metals.^50−57^ However, the application of the dual filtration method to specifically target bacteria capable of secreting metallophores is less studied. In our methodology, we employed two steps, the initial step involves the utilization of copper and cerium as a primary screening tool to identify bacteria that exhibit tolerance toward the applied heavy metals. Bacterial resistance to heavy metals can occur through different mechanisms, only one of which is the secretion of metallophores.^58,59^ Given this, the subsequent CAS assay is employed to allow the selection of bacteria secreting metallophores, specifically siderophores. Using this approach, we successfully isolated eight new metal-tolerant metallophore-producing bacteria. We validated the utility of this workflow by using LCMS-based metabolomics and genome mining studies of *C. basilensis* BL-MT-10 to reveal the production of taiwachelin within this strain, to our knowledge the first report of this molecule from this species. There are possibilities for refining this methodology, particularly in terms of isolating and identifying other metal-specific metallophore producers in light of the lack of observed copper binding by taiwachelin. A revised method could replace copper and cerium with different metals, or similarly, the second filtering step could be adapted by complexing the CAS dye with these alternative metals.^27,39,40,60^ In addition, we can utilize the MassQL tool to aid in the metabolomic identification of novel metal-bound small molecules.^45,61^ Future investigations will be needed to prove the hypothesized dual role of **1**, as both siderophore that aids in iron acquisition and a metallophore that sequesters toxic heavy metals. Given the lack of observed copper binding by **1**, investigations of the BGC regulation are needed to show if copper metal stress, the screen used to isolate *C. basilensis* BL-MT-10, induces taiwachelin production. We will also apply our isolation methods to the other bacterial isolates from this work to build a repository of novel metallophores, allowing investigations of their metal–metallophore interactions and thereby helping to identify candidates for heavy-metal bioremediation.

## Methods

### Sample Collection and Processing

In May 2022, soil samples were collected from the Carpenter Snow Creek Mining District, a Superfund site in Montana, United States. Samples were collected from surface soil within a depth of 20 cm, directly transferred to 50 mL sterile Falcon tubes, and preserved at 4 °C until processing. To extract the soil bacteria, approximately 0.5 g of soil from each sample was added to 1 mL of sterile phosphate-buffered saline and vortexed at room temperature for 5 min at maximum speed. Subsequently, the samples were allowed to stand undisturbed for an additional 5 min, facilitating the settling of any solid particles. Following this sedimentation step, the supernatants containing suspended microbial cells were used in the next step to inoculate the metal-toxified solid agar plates.

### Enrichment on Metal-Toxified Plates

50 μL of each sample was spread by a sterile glass plate spreader and allowed to grow on 15% agar-solidified plates of 1/5 LB, ISP-4, or DMS. Those media (1/5 LB, ISP-4, and DMS) were supplemented with 5 mg/L of various d-amino acids (d-valine, d-methionine, d-leucine, d-phenylalanine, d-threonine, and d-tryptophan obtained as high-purity compounds from different suppliers) as described by Nguyen and colleagues.^63^ A trace-metal solution of H_3_BO_3_, MnCl_2_·4H_2_O, ZnSO_4_·7H_2_O, Na_2_MoO_4_·2H_2_O, CuSO_4_·5H_2_O, and Co(NO_3_)_2_·6H_2_O, as in BG-11 medium,^64^ was also added (1 mL/L) to the culture media. Each medium was also toxified with either copper(II) chloride or cerium(III) chloride at concentrations of 2.5 or 5 mM, respectively. To mitigate the risk of water evaporation and ensure optimal conditions for bacterial growth, each plate was wrapped with parafilm and incubated at 28 °C. Metal-toxified plates were investigated daily to pick any colonies with different morphological features for further analysis. These colonies were picked directly into the next step, the CAS assay.

### CAS Screening

CAS plates were prepared as previously described by Louden and co-workers,^65^ with a slight modification including the utilization of either 1/5 LB or DMS as the base medium instead of the Minimal Media 9. Colonies picked from metal-toxified plates were recultured on their corresponding CAS medium (except for ISP-4 medium plates which were recultured on 1/5 LB-CAS plates as ISP-4 failed to form a stable blue color with the CAS dye). Plates were incubated at 28 °C and investigated for metal-binding small molecule secretion over 2 weeks. Bacteria that showed a positive yellow halo in the CAS assay were then picked and cultured on a liquid medium of either 1/5 LB or DMS with both d-amino acids and trace-metal supplementation as discussed before. After growth to turbidity, 25% glycerol stocks of those bacterial cultures were prepared by diluting an aliquot of the cultures 1:1 with sterile 50% glycerol and kept at −70 °C. These frozen stocks could be restarted by streaking out on plates for further investigations, as detailed below.

### DNA Extraction and 16S rRNA Gene Sequencing

For each bacterial isolate, approximately 5 mL of a fresh turbid liquid culture was used to extract DNA for 16S rRNA sequencing. DNA extraction was carried out using the OMEGA Bio-Tek E.Z.N.A. Bacterial DNA kit, following the manufacturer’s instructions without using the optional bead-beating step. Subsequently, the extracted DNA samples underwent 16S sequencing using the commercial vendor GENEWIZ’s Bacterial Identification Sanger-based service which targets V1 to V9.^66^ Vendor sequence data were then subjected to analysis using Geneious software, version 2021. Using Geneious software, sequences of our bacterial isolates were trimmed to remove high error portions of the Sanger runs and then compared against each other and against sequences within the GenBank public database.

### Whole Genome Sequencing and Genome Mining of *C. basilensis* BL-MT-10

HMW genomic DNA was isolated from the *C. basilensis* BL-MT-10 bacterium grown on 5 mL of DMS medium at 28 °C for 48 h. Following the incubation period, the bacterial cells were collected by centrifuging the entire culture broth at 21,000 rcf and 13 °C for 5 min. The bacterial pellet was subjected to HMW DNA isolation using the NucleoBond HMW DNA kit (Macherey-Nagel, Germany) following the manufacturer’s protocol with a modification in the lysis step. Briefly, the bacterial pellet underwent lysis using the bacterial cell lysis protocol as utilized in the OMEGA Bio-Tek E.Z.N.A. Bacterial DNA kit. For this step, TE buffer (100 μL) and lysozyme (10 μL) were added to the bacterial cell pellet, and this mixture was allowed to incubate for 10 min. Following this incubation period, an addition of TL buffer (100 μL) and proteinase K (20 μL) was made, followed by an hour-long incubation at 65 °C. Subsequently, 5 μL of RNase was introduced into the microcentrifuge tube and kept at room temperature for 5 min. After the lysis stage, the HMW DNA was isolated following the instructions detailed in the protocol of the NucleoBond HMW DNA kit. DNA was quantified using a Qubit fluorometer before shipping to the commercial vendor Plasmidsaurus for nanopore sequencing. The assembled genome obtained for *Cupriavidus basilenesis* was then subjected to the online genome mining software antiSMASH 7.0^48^ for its potential metallophore biosynthetic capacity.

### LCMS-Based Metabolomic Analysis of *C. basilensis* BL-MT-10

*C. basilensis* was grown on 2 × 5 mL of DMS medium for 3 days at 180 rpm and 28 °C. Clear supernatants were obtained by centrifuging the *C. basilensis* cultures for 10 min at 21,000 rcf and 13 °C. These supernatants were subsequently fractionated using a RP-SPE column (RP-SPE) following elution with 1000 μL each of Milli-Q H_2_O, 50% aqueous MeCN, and MeCN as eluting agents. The collected fractions were then subjected to LCMS analysis to screen for metallophores. We found that these metabolites were eluted in the 50% aqueous MeCN fraction. The LCMS system was equipped with a Core–Shell Kinetex, 2.6 μm 50 × 2.1 mm 100 Å EVO C18 column from Phenomenex. The LC gradient pump method employed 0.1% formic acid–acidified H_2_O (redistilled) as solvent A and 0.1% formic acid–acidified MeCN (LCMS grade, various suppliers) as solvent B. Our LCMS method for metallophore identification was used.^45^ Briefly, the gradient program consisted of an initial elution with 90% solvent A and 10% solvent B for 3 min, followed by a linear gradient to 25% solvent B at 5 min, further followed by a linear gradient to 99% solvent B over 7.5 min, with a 3 min hold at 99% solvent B, and finally a return to the initial elution conditions over 2 min, followed by a 2.5 min re-equilibration, all maintained at a flow rate of 450 μL/min. LCMS data analysis was conducted either manually or using the GNPS molecular networking tool to construct a molecular network.^49^ The network was generated with a Small Data Preset as a Networking Parameter, using specific settings including a minimum matched fragment ion value of 6, a minimum cluster size setting of 2, and a cosine score of 0.55. These data are available publicly from the MassIVE archive with accession ID: MSV000094901.

### Isolation of Taiwachelin Metallophore from *C. basilenesis* BL-MT-10

To isolate taiwachelin from the *C. basilensis* bacterium, a starter culture of 1 mL was introduced into 1 L of liquid DMS, divided into two separate batches, and placed in a 2.8 L baffled Erlenmeyer flask. The bacteria were allowed to grow at 28 °C with shaking at 180 rpm for 48 h. Subsequently, the metabolites were harvested from liquid cultures (2 × 1 L) by shaking with HP-20 resin (20 g/L) at 180 rpm for 2 h using an orbital shaker. The resulting suspension was filtered through filter paper to eliminate culture supernatant and cells, after which the remaining resin was rinsed with 1 × 500 mL of Milli-Q-purified water. The metabolites adsorbed to the resin were then eluted by using 4 × 100 mL of methanol. This methanol extract was concentrated via rotary evaporation, and the presence of metallophores was confirmed through LCMS analysis. The crude extract was subsequently fractionated using RP-SPE with a 5 g C18 column. Elution was carried out sequentially with 20 mL portions of H_2_O, 50% MeCN/H_2_O, and then MeCN. The fraction containing 50% MeCN/H_2_O was further purified via RP-HPLC (Agilent 1260 Infinity II HPLC with a multiple wavelength detector and a fraction collector), utilizing a C18 semipreparative column (Phenomenex Luna, 250 × 10 mm, 5 μm) with a flow rate of 3 mL/min. A gradient method of 0.1% formic acid–acidified H_2_O (Milli-Q) as solvent A and 0.1% formic acid–acidified MeCN (HPLC grade, Fisher Scientific) as solvent B was used. The gradient from 45% to 75% B over 15 min facilitated the isolation of the pure compounds. HPLC flowthrough was collected in 1 mL volumes, and peaks were identified by a 210 nm chromatogram. The organic solvent was removed from the collected fractions by rotary evaporation, and subsequently, the remainder was frozen and dried using a freeze-dryer (a Labconco Dry System/FreeZone 2.5 lyophilizer). The pure sample was then subjected to NMR and LCMS analyses. The NMR data sets are publicly available at the Natural Products Magnetic Resonance Database under archive number: NP0333454.

### Assessment of Taiwachelin Metal Binding

2.2 mg portion of isolated lyophilized taiwachelin was diluted into 221 μL of redistilled Milli-Q water (a 10 mM solution). Separately, metal salts, FeCl_3_·6H_2_O (Sigma-Aldrich, ACS grade), ZnCl_2_ (Fisher, ACS grade), CeCl_3_·6H_2_O (Aldrich 99.9%), and CuCl_2_·2H_2_O (Sigma-Aldrich), were also prepared as 10 mM stocks in Milli-Q water. An aliquot of the metallophore was mixed with the metal salt, and this mixture was diluted to 200 nM in 10% acetonitrile in water. These mixtures were run on LCMS with the method described above.

### Safety Considerations

Novel colonies from our strain isolation efforts were worked with in a Labconco Purifier Class I Safety Enclosure until 16S sequencing identified their nearest relatives as Biosafety Level 1 (BSL-1) organisms, at which point they were treated as such. Any group replicating our workflow should also treat uncharacterized bacteria as BSL-2, unless shown otherwise.

## Data Availability

The 16S rRNA data of the bacterial isolates were uploaded to GenBank under accession numbers PP868352–PP868368. The whole genome sequence of *C. basilensis* BL-MT-10 is available under the following BioProject number PRJNA1140264 with the accession numbers CP163419, CP163420, and CP163416–CP163418 assigned to our assembly of the chromosome, chromid, and plasmids, respectively. The LCMS data for the crude extract of *C. basilensis* BL-MT-10 used for metabolomic analysis and taiwachelin isolation have been uploaded to the GNPS-MassIVE archive with accession ID: MSV000094901. Raw NMR data has been submitted to the Natural Products Magnetic Resonance Database (NP-MRD) (https://np-mrd.org) and available with the following ID for compound **1**: NP0333454.

## Supporting Information Available

MS instrument details, MS and NMR data, and genomic analysis (PDF)
