# Supplementary material for: Dual Screen for Metal-Tolerant Metallophore Producers Evaluated with Soil from the Carpenter Snow Creek Site, a Heavy-Metal-Toxified Site in Montana
Source: ACS Omega. 2024 Dec 16;9(52):51213–20. doi: 10.1021/acsomega.4c07306 (PMC11696751; doi:10.1021/acsomega.4c07306)
Supplement: Supplementary file 1 — ao4c07306_si_001.pdf [file ao4c07306_si_001.pdf]

SUPPLEMENTARY INFORMATION

A Dual Screen for Metal-Tolerant Metallophore Producers Evaluated with Soil from the Carpenter-Snow Creek Site, a Heavy Metal-Toxified Site in Montana

Mohammed M. A. Ahmed[1,2†] Cameron Hammers[1] and Paul D. Boudreau[1*]

[1]Boudreau Lab, Department of BioMolecular Science, School of Pharmacy, University of Mississippi; Faser Hall, University, MS, USA, 38677-1848
[2]Department of Pharmacognosy, Al-Azhar University; Al-Azhar University, Nasr City, Cairo, Egypt, 11371

[†] Current affiliation: Department of Chemistry, University of Florida; PO Box 117200, Gainesville, Florida, USA

[*] Corresponding Author: E-mail boudreau@olemiss.edu (Paul D. Boudreau)

Table of Contents

Section S1. Supporting Information……………………………………………....………**S3-S10**

Table S1. List of Metallophore Producing Bacterial Isolates…………..……….........**S3**

Figure S1. OAT Analysis of *Cupriavidus basilensis* BL-MT-10……….……………..**S4**

Figures S2-3. HRMS Spectra and MS^2 Fragmentation Analysis of Taiwachelin (**1**) …......**S5**

Figures S4-5. HRMS Spectra and MS^2 Fragmentation Analysis of Taiwachelin Analog (**2**)…………………………………………………………………..……**S6**

Figures S6-8. ^1H, DEPTQ-^{13}C, HSQC spectra of Taiwachelin ………..……..…..….....**S7-S9**

Table S2. Comparison of ^{13}C-NMR of Reported Taiwachelin with Compound (**1**)....**S10**

Supplemental References. ……….……………………………...…………………………....**S11**

Section S1

Table S1: List of Metallophore Producing Bacterial Isolates

Strain Code	Accession Number	Nearest BLAST 16S Hit (Accession No.)	% Similarity	Read Length	Medium	Metal
BL-MT-01	PP868352	*Paraburkholderia calendonica* LMG 19076 (NR-025057)	98.6	844	DMD	$CeCl_3$
BL-MT-02	PP868353	*Paraburkholderia calendonica* LMG 19076 (NR-025057)	98.6	839	DMD	$CeCl_3$
BL-MT-03	PP868354	*Rhodanobacter denitrificans* 2APBS1 (NR-108437)	98.9	702	1/5 LB	$CuCl_2$
BL-MT-04	PP868355	*Rhodanobacter denitrificans* 2APBS1 (NR-108437)	98.3	847	1/5 LB	$CuCl_2$
BL-MT-05	PP868356	*Rhodanobacter denitrificans* 2APBS1 (NR-108437)	98.3	839	1/5 LB	$CuCl_2$
BL-MT-06	PP868357	*Dyella ginsengisoli* Gsoil 3046 (NR-041370)	99.6	247	ISP-4	$CuCl_2$
BL-MT-07	PP868358	*Bradyrhizobium erythrophlei* CCBAU 53325 (NR-135877)	100	1189	ISP-4	$CuCl_2$
BL-MT-08	PP868359	*Rhodanobacter umsongensis* GR24-2 (NR-108435)	98.2	1209	1/5 LB	$CuCl_2$
BL-MT-09	PP868360	*Luteibacter rhizovicinus* LJ96 (NR-042197)	100	1263	1/5 LB	$CuCl_2$
BL-MT-10	PP868361	*Cupriavidus basilensis* DSM 11853 (NR-025138)	99.9	928	DMD	$CuCl_2$
BL-MT-11	PP868362	*Arthrobacter gyeryongensis* DCY72 (NR-133699)	99.7	1167	1/5 LB	$CeCl_3$
BL-MT-12	PP868363	*Luteibacter rhizovicinus* LJ96 (NR-042197)	100	1216	1/5 LB	$CuCl_2$
BL-MT-13	PP868364	*Bradyrhizobium erythrophlei* CCBAU 53325 (NR-135877)	100	1225	ISP-4	CuCl2
BL-MT-14	PP868365	*Paraburkholderia fungorum* LMG 16225 (NR-025058)	100	1322	ISP-4	CuCl2
BL-MT-15	PP868366	*Paraburkholderia fungorum* LMG 16225 (NR-025058)	100	1169	ISP-4	CuCl2
BL-MT-16	PP868367	*Arthrobacter gyeryongensis* DCY72 (NR-133699)	99.7	1181	1/5 LB	CeCl3
BL-MT-17	PP868368	*Paraburkholderia fungorum* LMG 16225 (NR-025058)	100	1124	ISP-4	$CuCl_2$

Figure S1: OAT analysis of *Cupriavidus basilensis* BL-MT-10 against other *Cupriavidus* genus members

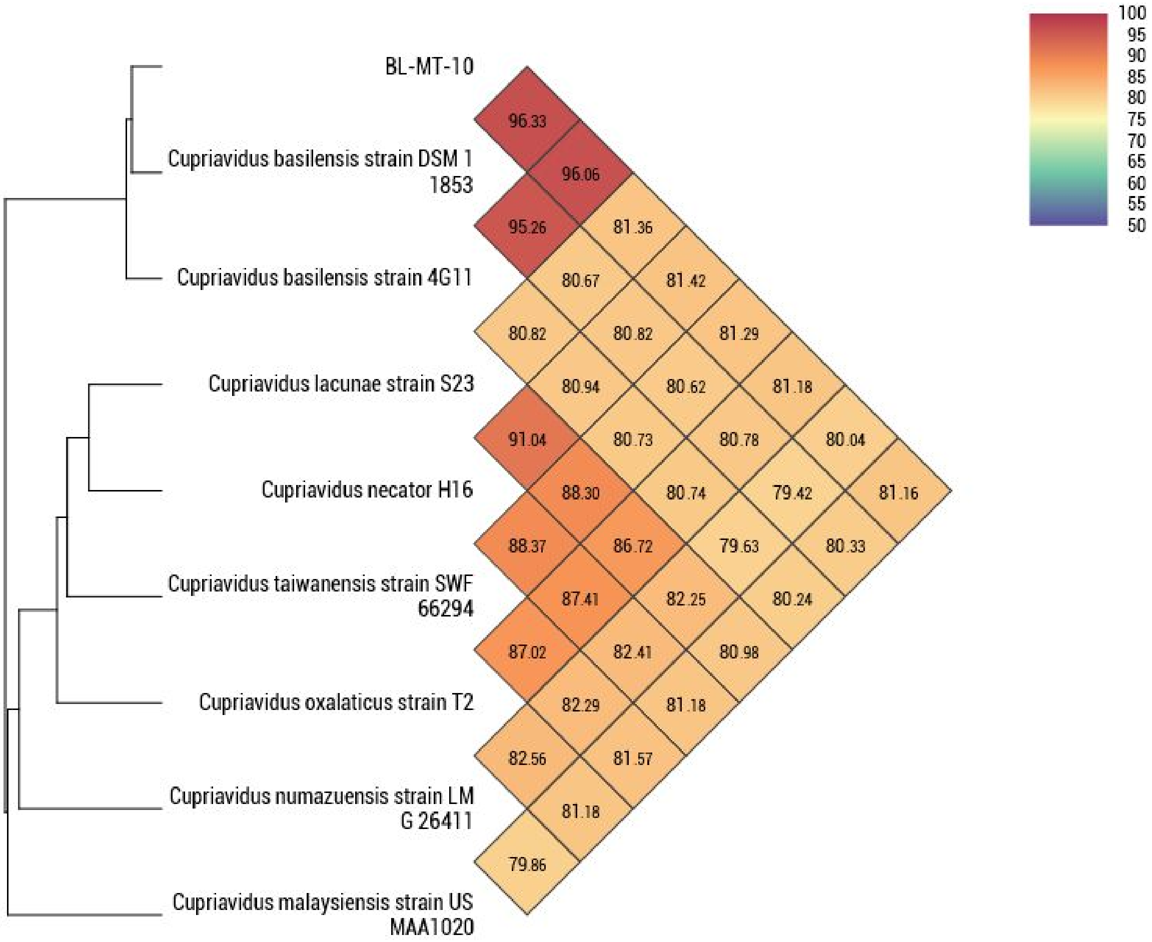

Heatmap and image generated using the OrthoANI tool.[1]

Figure S2: HRMS² Fragment Spectrum of Taiwachelin (1)

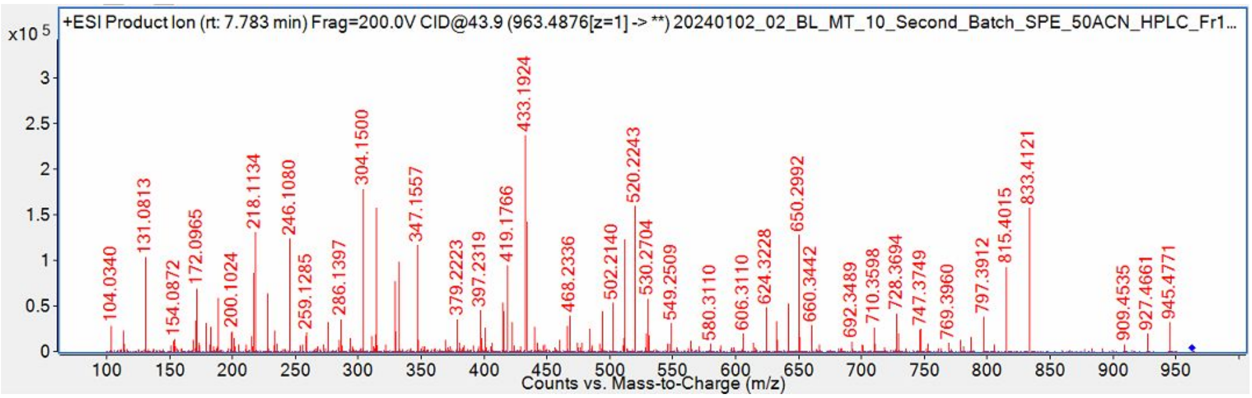

Figure S3: Fragment Structures of Taiwachelin (1)

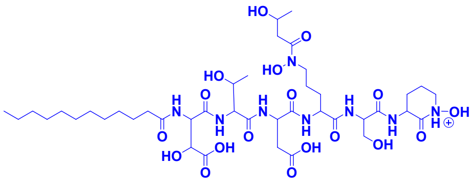

Chemical Formula: $C_{41}H_{71}N_8O_{18}^+$,
Exact Mass: 963.4881, Observed 963.4876,
error 0.5 ppm

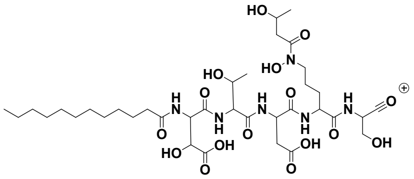

Chemical Formula: $C_{36}H_{61}N_6O_{16}^+$
Exact Mass: 833.4139, Observed 833.4121,
error 2.1 ppm

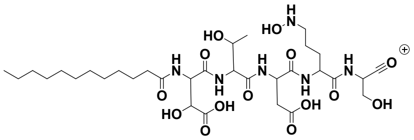

Chemical Formula: $C_{32}H_{55}N_6O_{14}^+$
Exact Mass: 747.3771, Observed 747.3749,
error 2.9 ppm

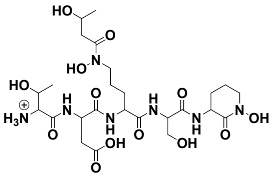

Chemical Formula: $C_{25}H_{44}N_7O_{13}^+$
Exact Mass: 650.2992, Observed 650.2992,
error 0.0 ppm

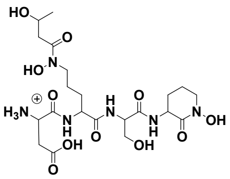

Chemical Formula: $C_{21}H_{37}N_6O_{11}^+$
Exact Mass: 549.2515, Observed 549.2509,
error 1.0 ppm

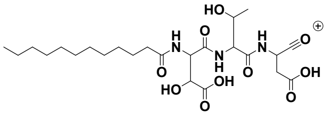

Chemical Formula: $C_{24}H_{40}N_3O_{10}^+$
Exact Mass: 530.2708, Observed 530.2704,
error 0.8 ppm

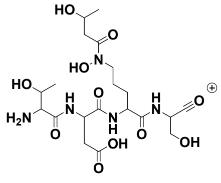

Chemical Formula: $C_{20}H_{34}N_5O_{11}^+$
Exact Mass: 520.2249, Observed 520.2243,
error 1.2 ppm

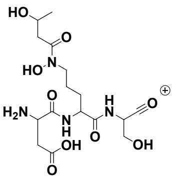

Chemical Formula: $C_{16}H_{27}N_4O_9^+$
Exact Mass: 419.1773, Observed 419.1766,
error 1.7 ppm

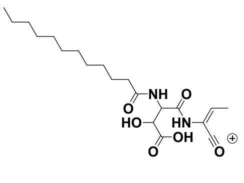

Chemical Formula: $C_{20}H_{33}N_2O_6^+$
Exact Mass: 397.2333, Observed 397.2319,
error 3.5 ppm

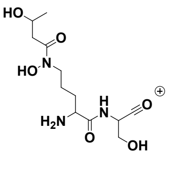

Chemical Formula: $C_{12}H_{22}N_3O_6^+$
Exact Mass: 304.1503, Observed 304.1500,
error 1.0 ppm

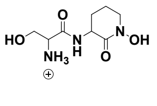

Chemical Formula: $C_8H_{16}N_3O_4^+$
Exact Mass: 218.1135, Observed 218.1134,
error 0.5 ppm

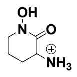

Chemical Formula: $C_5H_{11}N_2O_2^+$
Exact Mass: 131.0815, Observed 131.0813,
error 1.5 ppm

Figure S4: HRMS^2 Fragment Spectrum of Taiwachelin Analog (2)

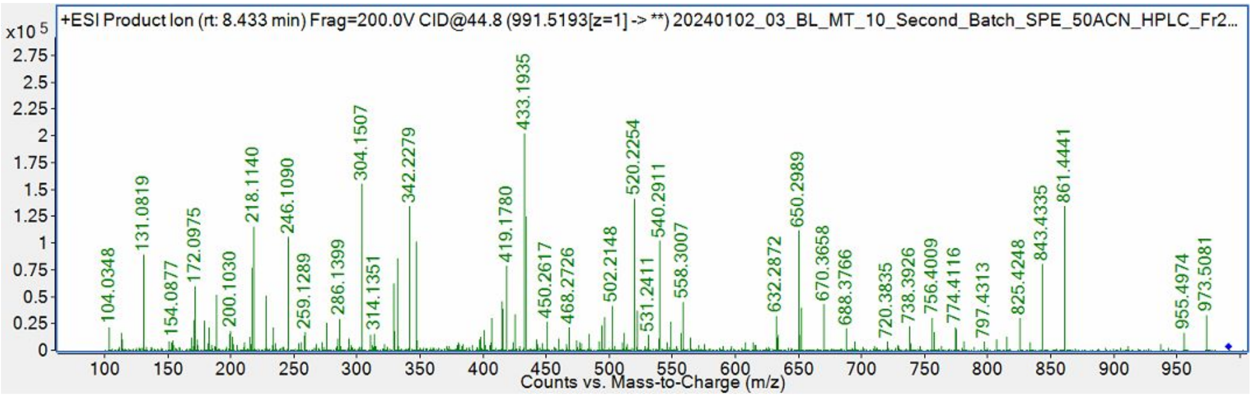

Figure S5: Fragment Structures of Taiwachelin Analog (2)

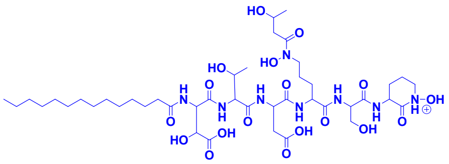

Chemical Formula: $C_{43}H_{75}N_8O_{18}^+$
Exact Mass: 991.5194, Observed 991.5193,
error 0.1 ppm

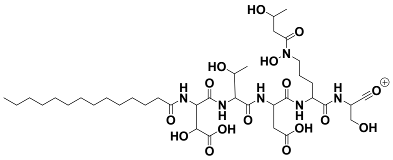

Chemical Formula: $C_{38}H_{65}N_6O_{16}^+$
Exact Mass: 861.4452, Observed 861.4441,
error 1.3 ppm

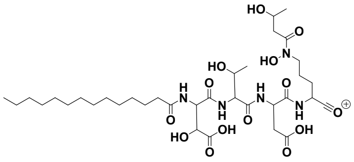

Chemical Formula: $C_{35}H_{60}N_5O_{14}^+$
Exact Mass: 774.4131, Observed 774.4116,
error 1.9 ppm

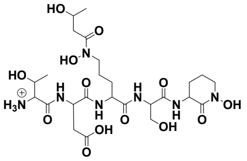

Chemical Formula: $C_{25}H_{44}N_7O_{13}^+$
Exact Mass: 650.2992, Observed 650.2989,
error 0.5 ppm

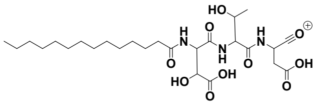

Chemical Formula: $C_{26}H_{44}N_3O_{10}^+$
Exact Mass: 558.3021, Observed 558.3007,
error 2.5 ppm

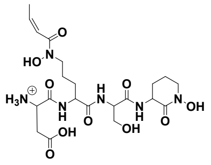

Chemical Formula: $C_{21}H_{35}N_6O_{10}^+$
Exact Mass: 531.2409, Observed - 0.4 ppm

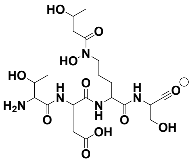

Chemical Formula: $C_{20}H_{34}N_5O_{11}^+$
Exact Mass: 520.2249, Observed 520.2254,
error - 1.0 ppm

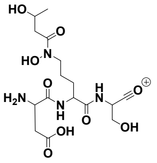

Chemical Formula: $C_{16}H_{27}N_4O_9^+$
Exact Mass: 419.1773, Observed 419.1780,
error - 1.7 ppm

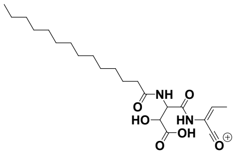

Chemical Formula: $C_{22}H_{37}N_2O_6^+$
Exact Mass: 425.2646, Observed 425.2643,
error 0.7 ppm

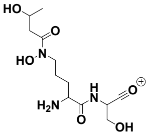

Chemical Formula: $C_{12}H_{22}N_3O_6^+$
Exact Mass: 304.1503, Observed 304.1507,
error - 1.3 ppm

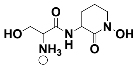

Chemical Formula: $C_8H_{16}N_3O_4^+$
Exact Mass: 218.1135, Observed 218.1140,
error - 2.3 ppm

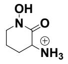

Chemical Formula: $C_5H_{11}N_2O_2^+$
Exact Mass: 131.0815, Observed 131.0819,
error - 3.0 ppm

Figure S6: ^{1}H-NMR (500 MHz, CD_3OD) Spectrum of 1

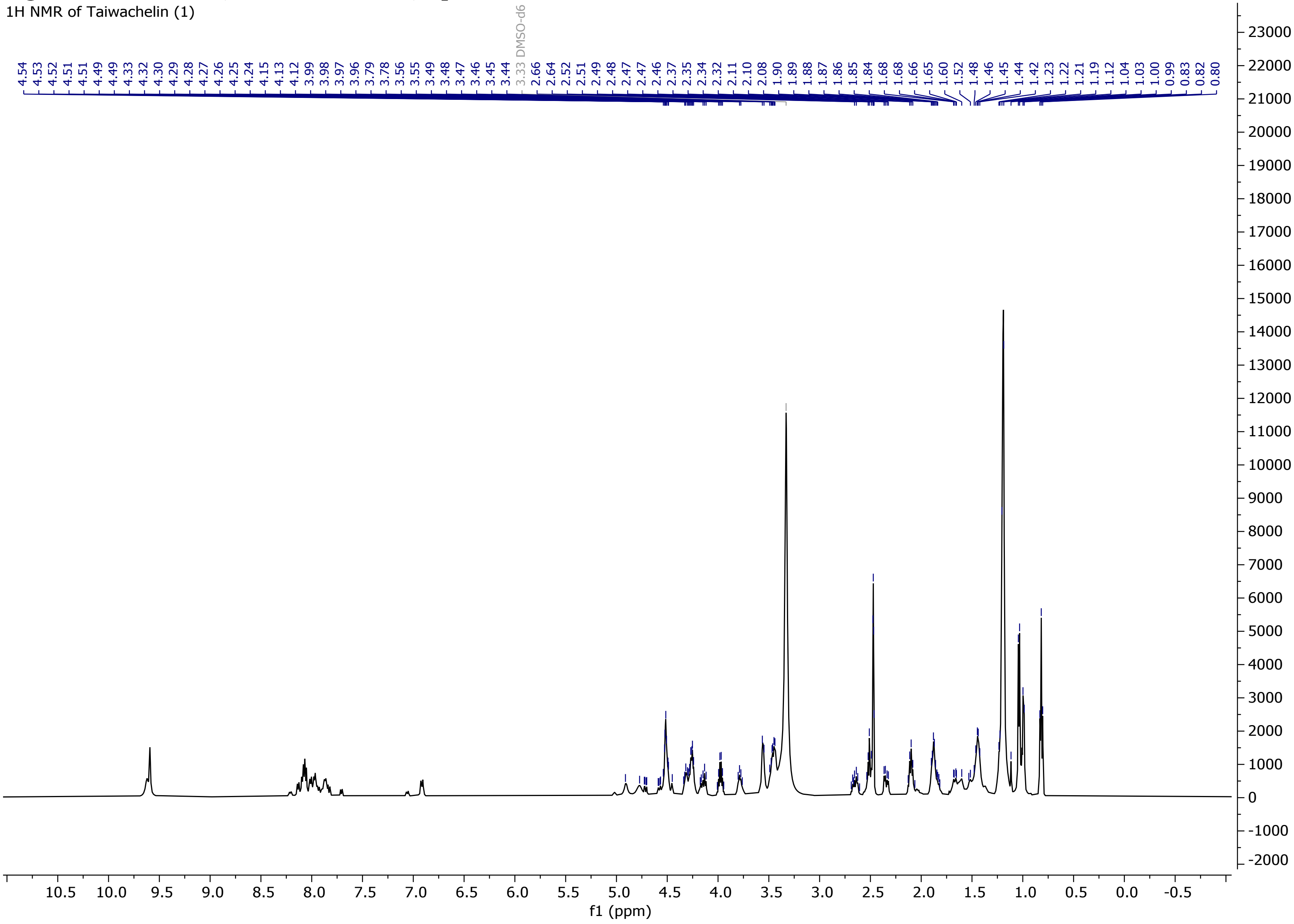

Figure S7: Dept-Q ^{13}C-NMR (125 MHz, CD_3OD) Spectrum of 1

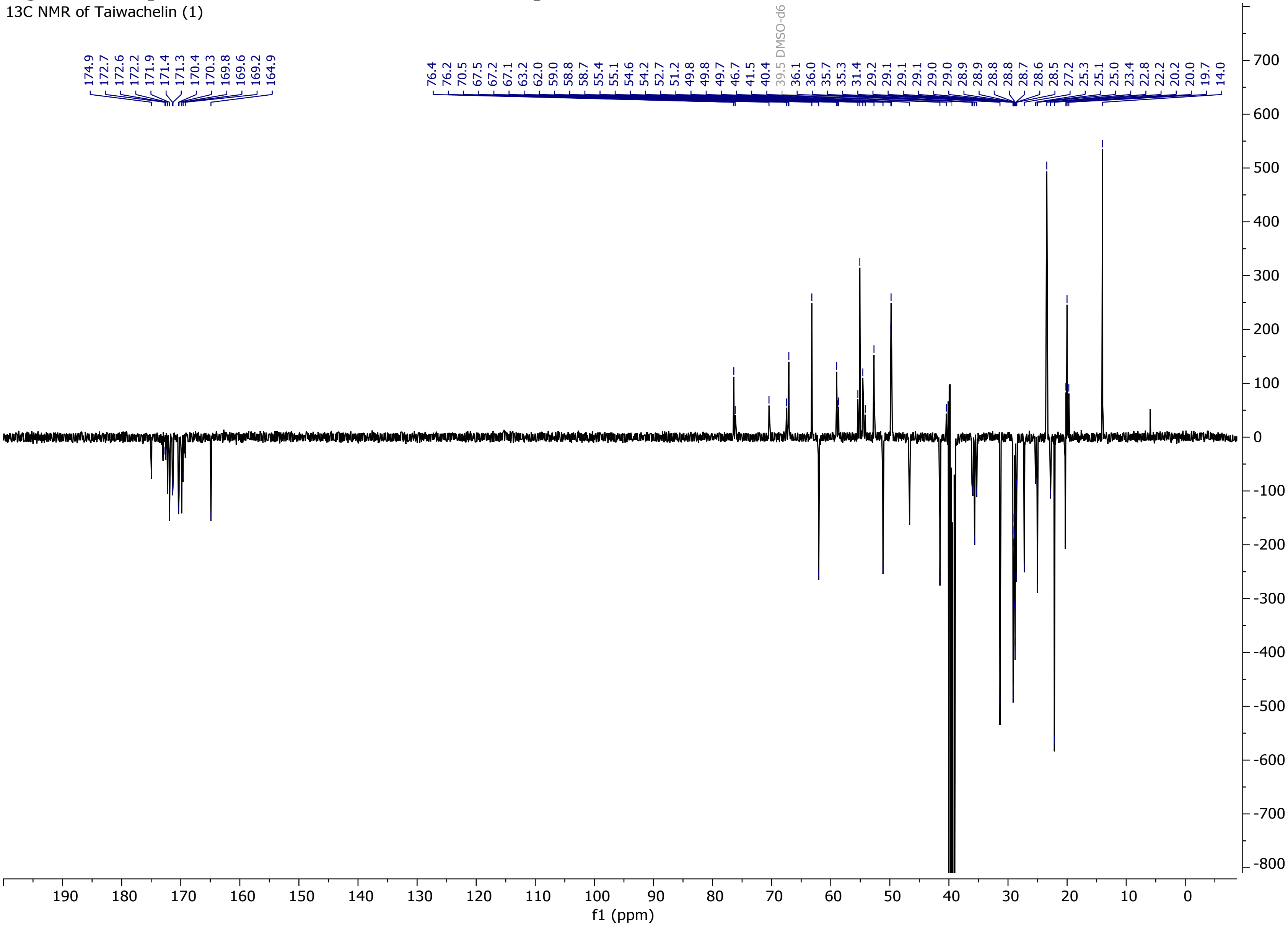

Figure S8: HSQC Spectrum of 1

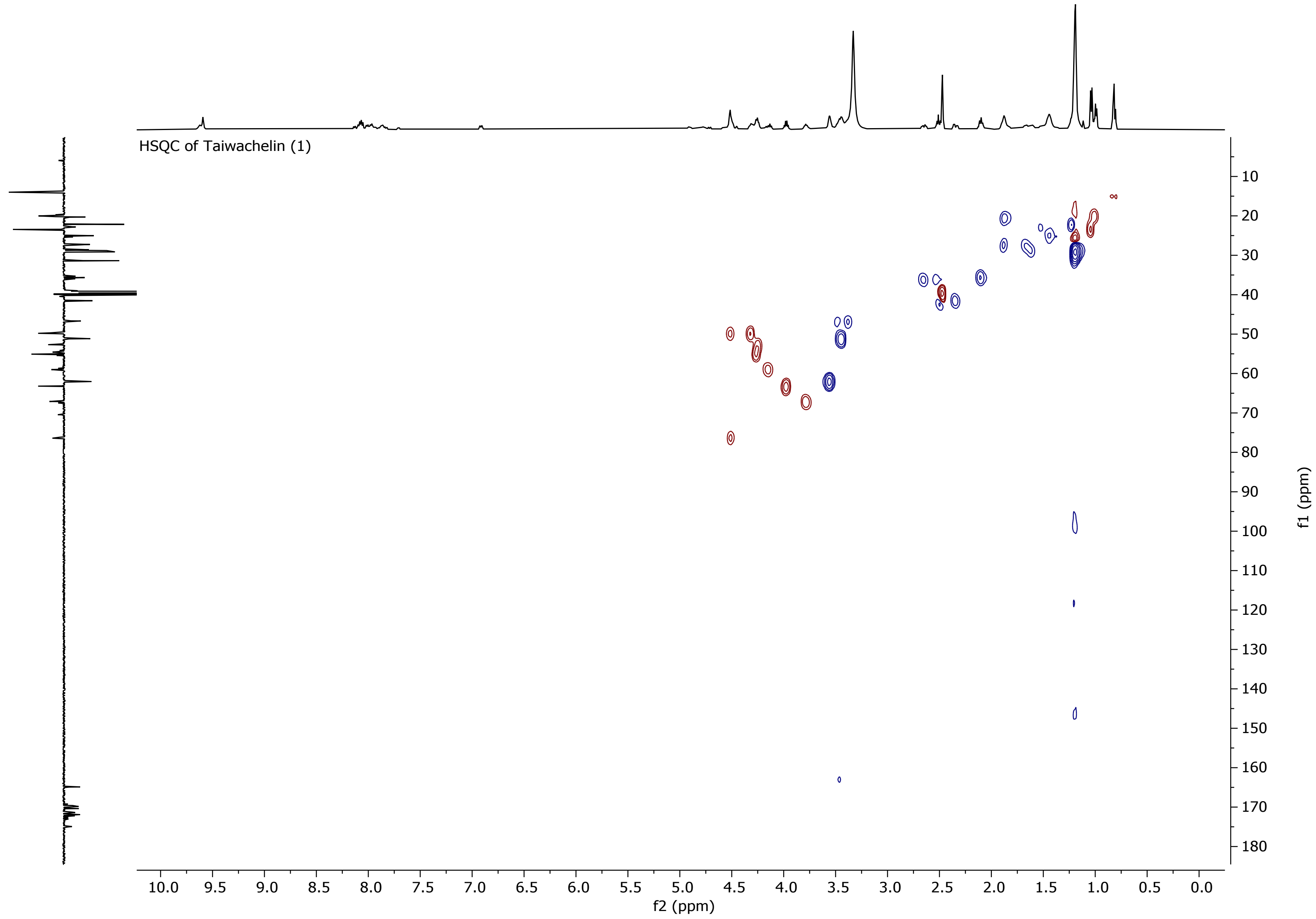

Table S2: NMR shift comparison of reported taiwachelin[2] and compound 1 (this study).

Residue	Position	δ_C, Taiwachelin	1δ_C, Compound **1**	Difference
Fatty acid	C-1	172.7	172.7	0.0
	C-2	35.3	35.3	0.0
	C-3	25.3	25.3	0.0
	C-4	28.7	28.7	0.0
	C-5	28.8	28.8	0.0
	C-6	29.0	29.0	0.0
	C-7	29.1	29.1	0.0
	C-8	29.1	29.1	0.0
	C-9	28.9	28.9	0.0
	C-10	31.4	31.4	0.0
	C-11	22.2	22.2	0.0
	C-12	14.0	14.0	0.0
L-*threo*-*β*-OH-Asp	CO	169.4	169.4	0.0
	Cα	55.5	55.4	0.1
	Cβ	70.5	70.5	0.0
	Cγ	173.0	172.9	0.1
D-*allo*-Thr	CO	170.0	170.0	0.0
	Cα	58.8	58.8	0.0
	Cβ	67.2	67.2	0.0
	Cγ	19.7	19.7	0.0
L-Asp	CO	170.3	170.3	0.0
	Cα	49.7	49.7	0.0
	Cβ	36.1	36.1	0.0
	Cγ	171.9	171.9	0.0
D-N^{δ}-OH-Orn	CO	171.4	171.4	0.0
	Cα	52.7	52.7	0.0
	Cβ	29.1	29.1	0.0
	Cγ	22.8	22.8	0.0
	Cδ	46.7	46.7	0.0
L-Hbu	C-1	171.4	171.4	0.0
	C-2	41.5	41.5	0.0
	C-3	63.2	63.2	0.0
	C-4	23.4	23.4	0.0
L-Ser	CO	169.9	169.8	0.1
	Cα	55.1	55.1	0.0
	Cβ	62.1	62.0	0.1
L-N^{δ}-OH-Orn (cyclic)	CO	164.9	164.9	0.0
	Cα	49.9	49.8	0.1
	Cβ	27.3	27.2	0.1
	Cγ	20.3	20.3	0.0
	Cδ	51.2	51.2	0.0
			Mean Difference:	**0.01**

References:

(1) Lee, I.; Kim, Y. O.; Park, S. C.; Chun, J. OrthoANI: An Improved Algorithm and Software for Calculating Average Nucleotide Identity. *Int. J. Syst. Evol. Microbiol.* **2016**, *66* (2), 1100–1103. https://doi.org/10.1099/ijsem.0.000760.

(2) Kreutzer, M. F.; Nett, M. Genomics-Driven Discovery of Taiwachelin, a Lipopeptide Siderophore from Cupriavidus Taiwanensis. *Org. Biomol. Chem.* **2012**, *10* (47), 9338–9343. https://doi.org/10.1039/c2ob26296g.
